# TRPV1 Receptor Identification in Bovine and Canine Mitral Valvular Interstitial Cells

**DOI:** 10.3390/vetsci8090183

**Published:** 2021-09-04

**Authors:** Cristina Vercelli, Graziana Gambino, Michela Amadori, Giovanni Re, Eugenio Martignani, Rossella V. Barberis, Izabela Janus, Massimiliano Tursi

**Affiliations:** 1 Department of Veterinary Sciences, University of Turin, 10095 Turin, Italy; graziana.gambino@unito.it (G.G.); giovanni.re@unito.it (G.R.); eugenio.martignani@unito.it (E.M.); massimiliano.tursi@unito.it (M.T.); 2 Life and Device S.r.l., 10095 Turin, Italy; rossella.barberis@lifeanddevice.com; 3 Department of Pathology, Wroclaw University of Environmental and Life Sciences, 50-376 Wrocław, Poland; izabela.janus@upwr.edu.pl

**Keywords:** Transient Receptor Vanilloid 1, valvular interstitial cells, mitral valve degeneration, immunohistochemistry, immunocytochemistry, bovine, canine

## Abstract

Myxomatous mitral valve degeneration (MMVD) is the most common acquired cardiac disease in canine species, and valvular interstitial cells (VICs) are considered the main responsible for the development of this pathology. The scientific interest is focused on isolating and characterizing these cells. The aims of the present study were to verify a novel VICs mechanical isolation method and to characterize isolated cells using immunocytochemistry and immunofluorescence, with parallel histological and immunohistochemistry assays on bovine and canine healthy and MMVD mitral valves. Antibodies against vimentin (VIM), smooth muscle actin (SMA), von Willebrand (vW) factor, Transforming Growth Factor (TGF) β1, and Transient Receptor Potential Vanilloid 1 (TRPV1) were used. The isolation method was considered reliable and able to isolate only VICs. The different assays demonstrated a different expression of SMA in healthy and MMVD mitral valves, and TRPV1 was isolated for the first time from bovine and canine VICs and the correspondent mitral valve leaflets. The novelties of the present study are the new isolation method, that may allow correlations between laboratory and clinical conditions, and the identification of TRPV1, which will lead to further investigations to understand its function and possible role in the etiology of MMVD and to the design of new therapeutic strategies.

## 1. Introduction 

Myxomatous mitral valve degeneration (MMVD) is the most common acquired cardiac disease in elderly dogs and causes high mortality due to cardiac insufficiency [1]. Canine MMVD presents similarities with the human disease and results predominantly in valvular insufficiency, blood regurgitation, subsequent left-sided volume overload, and finally, congestive heart failure [2,3]. Dogs affected by the disease may have a long preclinical period that could last for years prior to developing clinical signs, with progressive worsening of the pathology [4,5,6].

It has been demonstrated that valvular interstitial cells (VICs) are the main cell population in mitral valve leaflets in humans and respond to mechanical, chemical, and molecular changes that can damage leaflet integrity [7,8]. These cells are mainly involved in valvular homeostasis, preserving modifications of the extracellular matrix (ECM), composed of collagen, elastin, and proteoglycans [9]. VICs phenotype can be heterogenous, presenting differences in cell morphology and function that can vary along the lifetime and can lead to a pathological process [10]. The transition from quiescent or normal to activated VIC is reflected by a morphology change from a fibroblasts-like to a myofibroblast phenotype, with double immunoreactivity for vimentin (VIM) and smooth muscle actin (SMA) [7,11]. Myofibroblasts can promote deposition of proteoglycans and collagen fragmentation, compromising the structure of the coaptation margin on the atrialis aspect of the leaflet and causing loss of functionality [12,13]. Despite the huge interest in this topic, several questions about the triggering factors behind this activation remain unanswered. In the last years, several investigations were focused on understanding the mechanisms responsible for the activation. Some authors investigated the role of the interaction between VICs and valvular endothelial cells (VECs) both in 2D and in 3D models [14,15], others focused on the identification of serotine receptor (5HT) in VICs [2] of mechanoreceptors [8]. Previous research isolated VICs using enzymatic treatment (i.e., collagenase) and seeding, obtaining cells or small fragments of the mitral leaflet [7,16]. According to all the aforementioned factors and to the authors’ knowledge, no other isolation method has been tested, and no former investigations were focused on the identification of Transient Receptor Potential Vanilloid 1 (TRPV1) in VICs. TRPV1 has been identified in dogs [17] and previously characterized in canine keratinocytes and mammary cancer [18,19]. TRPV1 belongs to the complex family of TRP receptors, identified in human and veterinary species, with an unclear role in both physiological and pathological conditions [19,20]. It has been demonstrated that at least 25 members of TRPs family are expressed in cardiac tissue, mostly in cardiomyocytes: these receptors are permeable to Ca^2+^ and can modulate the excitability of cardiomyocytes, and their involvement in contraction and remodeling has been demonstrated [21]. Several TRPV1 agonists are routinely used for pain relief, but the potential use of TRPV1 analogues in the treatment of cardiovascular diseases is just emerging.

The aims of this work were: (i) to standardize and prove the soundness of the novel isolation method in a preliminary study using the bovine mitral valve; (ii) to characterize the isolated cells using immunocytochemistry; (iii) to apply the same procedure to healthy and pathological canine mitral valves; (iv) to characterize cells and to identify the presence of TRPV1 using immunocytochemistry, immunohistochemistry, and histology assays.

## 2. Materials and Methods

### 2.1. Method Standardization

Bovine hearts were collected in the teaching slaughterhouse of the Department of Veterinary Science of Turin (Turin, Italy). Fifteen animals aged 9 months were slaughtered for commercial purposes and not for experimental aims. All the procedures were performed under the supervision of a veterinarian of the Italian National Health Service and with the slaughterhouse chief’s consent. Hearts were collected within 1 h after death, washed with cold phosphate-buffered saline (PBS), and transported to the laboratory of the division of Pharmacology and Toxicology of the same Department, where the procedures were performed under sterile conditions and using a laminar flow hood.

External gross examination did not show any alteration. Hearts were opened along the caudal margin including both the left atrium and the left ventricle, thus obtaining a broad presentation of the mitral valve. Therefore, the mitral valves were examined by an expert cardiopathologist and were explanted by performing a dissection along the valvular annulus and the insertion of the papillary muscles in the ventricular wall. Briefly, the endocardial layer of the atrialis aspect of the last third of the leaflets was scraped using a scalpel blade, and then the subendothelial cell layer was scraped using a new scalpel blade. The harvested material was seeded in a 3 m-diameter Petri dish in the presence of complete culture medium, composed of Dulbecco’s modified Eagle’s medium (DMEM), 20% of Fetal Bovine Serum (FBS), 2% of l-glutamine, and 2% of antibiotic–antimycotic solution. Petri dishes were incubated at 37 °C in a 5% CO_2_ atmosphere and checked daily. The remaining part of the valves was fixed in 4% neutral buffered formaldehyde (NBF) for further histological and immunohistochemistry examinations. It was possible to appreciate fibroblast-like cells after 3–4 days. Immunocytochemistry (ICC) assays with anti-VIM, anti-SMA, and anti-von Willebrand (vW) factor antibodies were performed in triplicate. The results confirmed that only VICs were collected and seeded. Specific details about the procedures are explained in the next sections. 

### 2.2. Canine Samples

According to the encouraging results obtained using bovine mitral valves, two canine hearts were collected in the postmortem room of the Department of Veterinary Sciences of Turin (Turin, Italy). The two dogs were euthanized for different clinical conditions, not for experimental purposes. Owner consent was signed to give permission to the echocardiographic procedure, euthanasia, necropsy, and heart collection. The first dog was an 8-year-old female, Boxer breed, euthanized for a severe clinical condition consequently to a mammary tumor. Echocardiography did not highlight any abnormality, and the heart was enrolled as a normal healthy heart. The second dog was a 16-year-old mixed-breed, small-size, male dog presenting severe myxomatous mitral valve disease (MMVD) at the echocardiographic examination, with mitral regurgitation, rupture of chordae tendineae, and pulmonary oedema as observed by X-ray examination. It was classified as stage D2 according to the ranking of the American College of Veterinary Internal Medicine (ACVIM) [6]. 

Heart collection was performed within 4 h after death in both cases. The gross external and internal examination of the hearts was performed by an expert cardiopathologist. The valves were ranked using Whitney classification [22]: one was classified as class 1, in the absence of any alteration, while the heart collected from the dog with MMVD presented severe thickened and irregular valve leaflets and was classified as class 4. Moreover, that heart shown a severe dilation of the left atrium and ventricle, with several rupture of the chordae tendineae.

### 2.3. Cell Culture Method 

Mitral valves were placed on a platform to show the entire atrial aspect of both leaflets. The endothelial surface of the atrial aspect was scraped with a scalpel blade. After changing the blade, the atrialis layer was scraped, and the resulting subendocardial material was placed into uncoated 3 cm-diameter Petri dishes, filled with 3 mL of a complete and routinely used medium composed of high glucose Dulbecco’s modified Eagle’s medium (DMEM), 20% fetal bovine serum (FBS), 2% l-glutamine, and a 2% antibiotic–antimycotic solution (penicillin/streptomycin and amphotericin B). All reagents were purchased from Sigma Aldrich (Milan, Italy). Petri dishes were incubated at 37 °C with 5% CO_2_ and then checked daily. 

When reaching confluence (80–90%), cells were detached using a 1x trypsin solution (Sigma Aldrich, Milan, Italy) for 5 min, then buffered with fresh complete medium, and centrifugated. The supernatant was removed, and the pellet was split: some cells were seeded in 75 cm^3^ flasks with a vented screw cap (Nunc, Thermo Fisher Scientific, Waltham, MA, USA), while the remaining cells were stored at −80 °C with a cryopreserving solution composed of complete medium and 10% dimethyl sulfoxide (DMSO, Sigma Aldrich, Milan, Italy) for further experiments.

### 2.4. Immunocytochemistry Protocol

ICC assays were performed to characterize the cells and to ensure that only VICs were cultivated.

Cells were seeded at a density of 5 × 10^3^ cell/well on sterile round glass coverslips placed in 3 cm-diameter Petri dishes and cultured in the presence of complete medium, as described previously. Cells were incubated at 37 °C and 5% CO_2_ till they reached 80% of confluence. Coverslips were then rinsed twice in PBS, fixed in 4% NBF for 10 min at room temperature, and washed three times in PBS.

According to the method proposed by Freshney [23], an antibody for VIM was used to identify mesenchymal cells, an antibody for SMA was used to identify myofibroblasts, and an antibody for vW factor was used to identify the endothelium. The last one was chosen to confirm the absence of VECs. VIM, SMA, and vW factor antibodies were purchased from Dako Agilent (Santa Clara, CA, USA), while TGF β1 and TRPV1 antibodies were purchased from Santa Cruz Biotechnology Inc. (Dallas, TX, USA). A complete list of the antibodies and their optimal dilutions are presented in Table 1.

A standard ICC protocol using the Dako REAL™ EnVision™ Detection System, Peroxidase/DAB+ Rabbit/Mouse (cat. K 5007, Dako Agilent, Santa Clara, CA, USA) was used for ICC reactions according to the manufacturer’s instructions. Briefly, endogenous peroxidase activity was blocked using 2.5% H_2_O_2_ in methanol (Sigma Aldrich, Milan, Italy) at room temperature. The cells on coverslips were rinsed in PBS, permeabilized using 0.05% Tween-20 (Sigma Aldrich, Milan, Italy) in PBS, and finally treated with 1.5% bovine serum albumin (BSA, Sigma Aldrich, Milan, Italy) in PBS for 30 min to block non-specific binding. Thereafter, the cells were incubated for 48 h in a humidity chamber at 4 °C with primary antibodies against VIM, SMA, vW factor, TGF β1, and TRPV1, diluted in 1.5% BSA/PBS. The cells were then washed three times in PBS containing 0.05% Tween-20 and then processed using DAKO Envision^®^+ staining kit (Dako Inc., Carpinteria, CA, USA).

Negative controls underwent the same procedure with the omission of the primary antibody and consistently yielded negative results.

Different cell lines were used as positive controls according to the antibody of interest. All procedures were performed in triplicate.

### 2.5. Immunofluorescence Assay

The cells were cultivated as previously explained for the immunocytochemistry procedure. Then, they were incubated for 45 min in blocking buffer composed of 1.5% BSA, 0.05% Tween-20, PBS (Sigma Aldrich, Milan, Italy), at room temperature and then for 48 h in a humidity chamber at 4 °C with the primary antibodies (Table 1). The coverslips were washed twice with PBS and incubated with the appropriate secondary antibodies for 1 h at room temperature in a dark humid chamber.

Both primary and secondary antibodies were diluted in PBS containing 1.5% BSA. The samples were washed twice in PBS, then incubated in 1 µg/mL 4′,6-diamidino-2-phenylindole dihydrochloride (DAPI) (Sigma Aldrich, Milan, Italy) in PBS for 10 min, and mounted with ProLong Gold Antifade Reagent (Life Technologies, Monza, Italy).

The negative controls underwent the same procedure with the omission of the primary antibody and consistently yielded negative results. Different cell lines were used as positive controls according to the antibody of interest. All procedures were performed in triplicate.

The slides were examined using a Leica AF6000 LX system (Leica Microsystems CMS GmbH, Mannheim, Germany).

### 2.6. Histology and Immunohistochemistry Protocol

The mitral valve apparatus was fixed in 4% Neutral Buffered Formalin (NFB) for 48 h, then longitudinal portions of both leaflets, including the scraped areas, were sampled for histology. Sections of 3–5 µm thickness were stained with hematoxylin and eosin (H&E). Supplementary sections on poly-l-lysine-coated slides were processed for immunohistochemistry (IHC). The IHC technique was used to study the phenotypic characteristics of the valvular leaflets in comparison with cultured cells. A standard IHC protocol using the Dako REAL™ EnVision™ Detection System, Peroxidase/DAB+ Rabbit/Mouse (cat. K 5007, Dako Agilent, Santa Clara, CA, USA) was used. Following de-hydration and wax embedding, longitudinal sections were mounted on Menzel-Glaser Superfrost Plus slides (Thermo Scientific, Waltham, MA, USA). Slides were rehydrated, and endogenous peroxidase activity was blocked using 2.5% H_2_O_2_ in methanol (Sigma Aldrich, Milan, Italy) for 30 min at room temperature; the slides were then rinsed with distilled water for 5 min. To facilitate the immunostaining, antigen retrieval was performed for VIM, vW factor, TGF β1, and TRPV1. Slides were placed for 30 min in a thermostatic bath at 98 °C immersed in citrate buffer (pH = 6), except for those undergoing TGF β1 staining, which were immersed in Tris-EDTA (pH = 9).

After 15 min at room temperature, the slides were rinsed with distilled water, then washed three times for 5 min in a 0.05% Tween-20 solution in PBS.

The sections were finally incubated with 1.5% BSA in PBS for 30 min to block non-specific binding and incubated over-night at 4 °C with antibodies, using the dilutions listed in Table 1. The slides were then washed three times in PBS containing 0.05% Tween-20 and then processed using the DAKO Envision+ staining kit.

Negative controls underwent to the same procedure, omitting the primary antibody, and consistently yielded negative results. Vessel walls and endocardium present in the samples were considered as positive controls.

### 2.7. Development of Colorimetric Reactions of ICC and IHC

After a faint counterstaining with hematoxylin (Merck, Darmstadt, Germany), the specimens were dehydrated using alcoholic solutions with a progressively increased alcohol concentration, cleared in xylene (Sigma Aldrich, Milan, Italy), and mounted with DPX (Sigma Aldrich, Milan, Italy). All samples were examined using an Olympus BX40F4 microscope (Olympus Official, Tokyo, Japan) equipped with a Nikon Digital Sight DS-Fi1 camera (Nikon Corporation, Tokyo, Japan). Images were acquired with NIS-Elements F2.30 software (Nikon Corporation, Tokyo, Japan).

## 3. Results

### 3.1. Method Standardization

**Figure 1:**
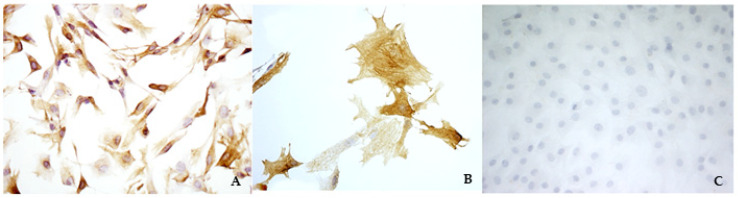
Immunocytochemistry analysis of bovine valvular interstitial cells (VICs) to visualize vimentin (**A**) (magnification: 10×), smooth muscle actin (**B**) (magnification: 40×), and von Willebrand factor (**C**) (magnification: 10×).

A preliminary study aimed to verify if this simplified technique was able to specifically isolate mitral valvular interstitial cells from bovine hearts. Viable and proliferating cells were obtained from 14 out of 15 bovine hearts collected at the slaughterhouse. Cells had a fibroblast-like morphology, and the immunocytochemistry evaluation confirmed that only VICs were isolated and cultured, as shown by a strong and diffuse cytoplasmatic positivity to VIM and a sparsely positivity to SMA (Figure 1A,B). The cells were negative to vW factor, which excluded the presence of VECs (Figure 1C).

### 3.2. Cell Cultures

Both bovine and canine VICs reached confluence at passage 0 (P0) within 8–10 days. Spindle-shaped cells, proliferating in culture, were evident after 2–3 days from seeding. The morphology was fibroblast-like both for bovine and for canine VICs, and no species-specific morphological characteristics were observed. In order to obtain a sufficient number of cells to perform the experiments, bovine VICs were used from passage 1 to passage 8, while canine VICs were used from passage 1 to passage 6. Subsequently, the proliferation rate decreased, and cell morphology changed from a spindle to a polygonal shape. For these reasons, it was decided to exclude these cells for further evaluations.

### 3.3. Immunocytochemistry and Immunofluorescence

For all cell cultures, ICC evaluation using VIM, SMA, and vW factor antibodies was performed to ensure that only VICs were cultured. The strong positivity to VIM confirmed the mesenchymal origin also for the cells isolated from the canine leaflets. No differences were highlighted among bovine cells and both healthy and pathological canine VICs.

**Figure 2:**
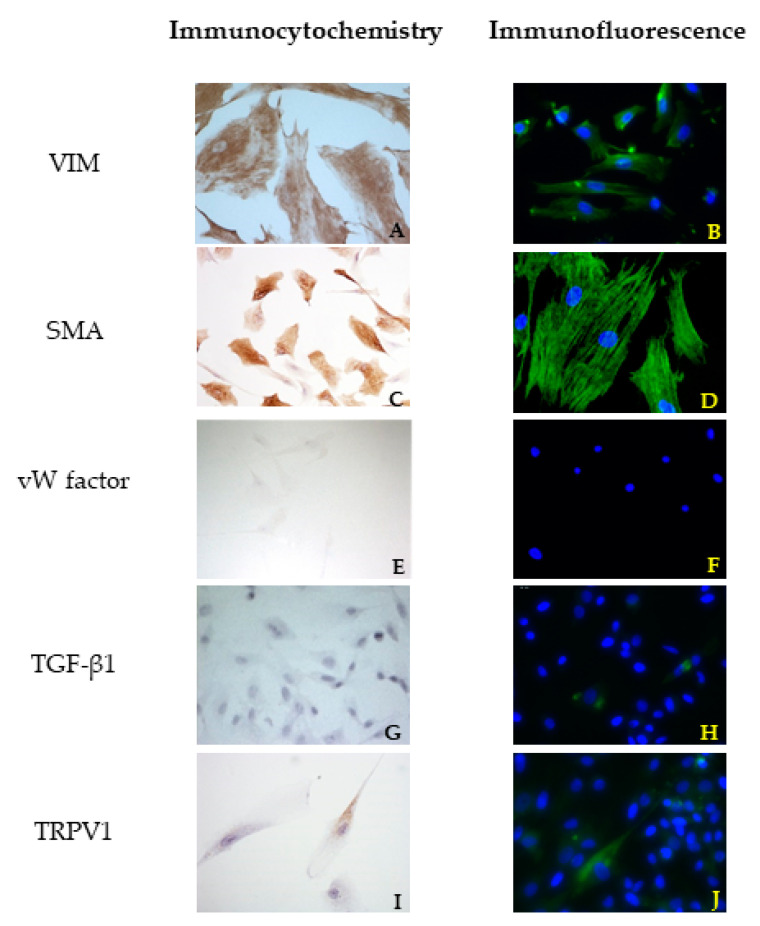
Results of immunocytochemistry (ICC) (**A**,**C**,**E**,**G**,**I**) and immunofluorescence (**B**,**D**,**F**,**H**,**J**) assays using vimentin (VIM), actin smooth muscle (SMA), von Willembrand (vW) factor, Trasforming growth Factor (TGF) β1, and Transient Receptor Potential Vanilloid 1 (TRPV1) antibodies in cells derived from a healthy mitral valve.

**Figure 3:**
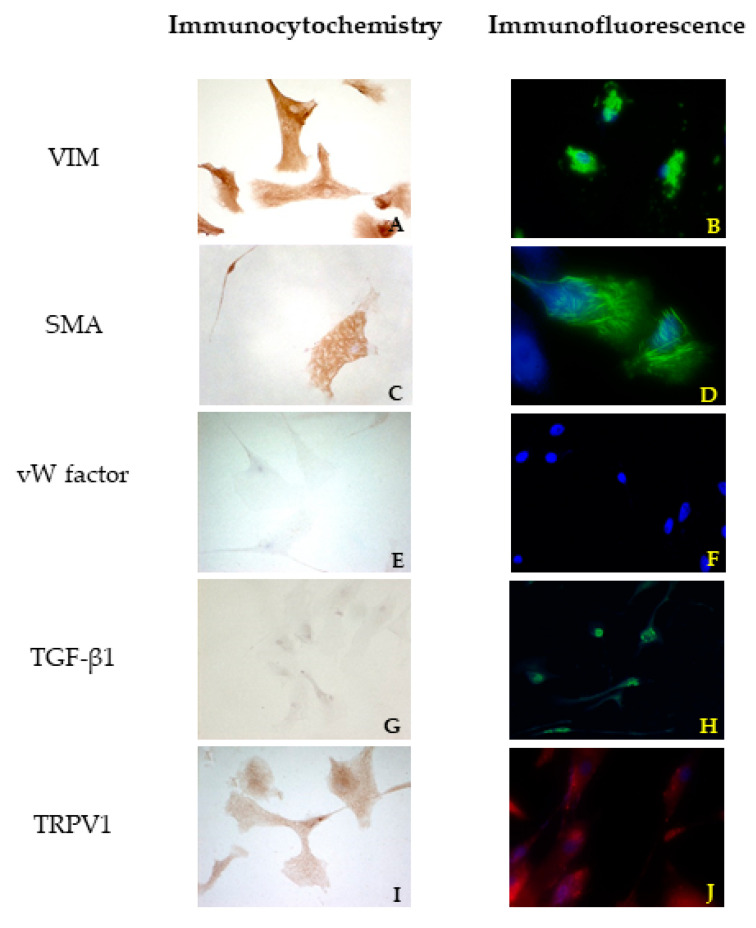
Results of immunocytochemistry (ICC) (**A**,**C**,**E**,**G**,**I**) and immunofluorescence (**B**,**D**,**F**,**H**,**J**) assays using VIM, SMA, vW factor, TGF β1, and TRPV1 antibodies in cells derived from an MMVD mitral valve.

In canine VICs, a different orientation of microfilaments of the cytoskeleton was observed in SMA reactions comparing healthy and diseased dog mitral valves. In the first case, the cells showed mostly a longitudinal orientation of microfilaments, while in cells obtained from the MMVD valve, microfilaments were orientated both longitudinally and transversally (Figure 2C and Figure 3C). No differences were highlighted between cells isolated from bovine and healthy canine valves. A further investigation about TGF β1 revealed a weak positivity in ICC for all cell lines (Figure 2G and Figure 3G). Immunofluorescence revealed a stronger positivity in pathological canine VICs compared to the physiological ones (Figure 2H and Figure 3H).

A granular cytoplasmatic positivity for TRPV1 was appreciated in all cell cultures in ICC. In order to confirm this result, cells were treated with a fluorescent antibody, and a strong cytoplasmatic positivity was highlighted.

Immunofluorescence confirmed the results obtained with ICC. An irregular orientation of the microfilaments positive to SMA was clearly observed in cells derived from pathological valves (Figure 2D and Figure 3D). TGF β1 reactions demonstrated a sparsely and week cytoplasmatic positivity in healthy cells (Figure 2H), while a more evident positivity was shown in MMVD-derived cells (Figure 3H). Immunofluorescence demonstrated a strong cytoplasmatic positivity to TRPV1 (Figure 2J and Figure 3J).

### 3.4. Histology and Immunohistochemistry

**Figure 4:**
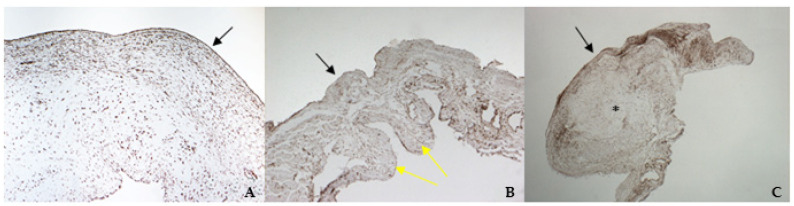
The picture shows positivity to vimentin (VIM) in bovine (**A**) (magnification: 5×) and healthy (**B**) (magnification: 5×) and pathological (**C**) (magnification: 5×) canine mitral valves. Black arrows indicate the atrial aspect, while yellow arrows indicate the origin of the cordae tendinae (ventricular aspect). *: area of myxomatous degeneration.

**Figure 5:**
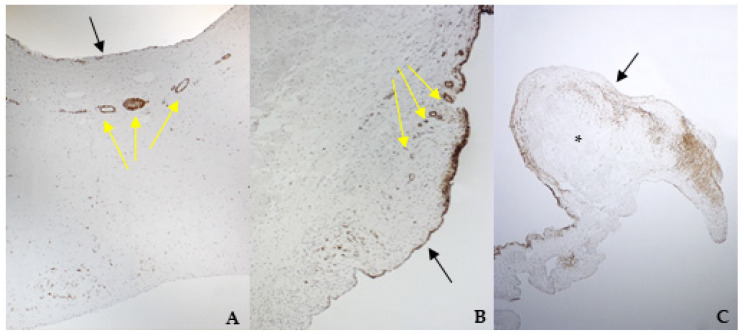
The picture shows positivity to smooth muscle actin (SMA) in bovine (**A**) (magnification: 5×) and healthy (**B**) (magnification: 10×) and pathological (**C**) (magnification: 5×) canine mitral valves. Black arrows indicate the atrial aspect, while yellow arrows indicate small arteries of the spongiosa layer. *: area of myxomatous degeneration.

**Figure 6:**
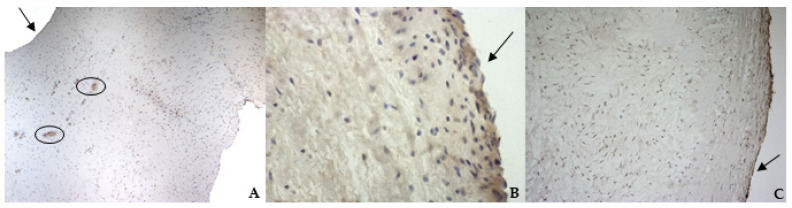
The picture shows positivity to von Willebrand (vW) factor in the endocardial layer of bovine (**A**) (magnification: 5×) and healthy (**B**) (magnification: 10×) and pathological (**C**) (magnification: 10×) canine mitral valves. Black arrows indicate the atrial aspect. In (A), black circles delimitate small arteries in the spongiosa layer.

The histological examination of bovine and healthy canine mitral valves showed a normal stratified structure with a regular conformation of collagen (Figure 4A,B). Instead, the valve leaflets of the dog enrolled as pathological, presented severe myxomatous degeneration characterized by multifocal proliferation of spindle cells mixed to abundant eosinophilic intercellular matrix in the spongiosa and fibrosa layers, with collagen fragmentation. These cells demonstrated an heterogenous pattern, with central nuclei and scant homogeneous cytoplasm (Figure 4C and Figure 5C). IHC confirmed myxomatous degeneration in large areas of the leaflets, as shown by the double immunoreactivity to VIM and SMA of the spindle cells. The endocardial cells lining the valve leaflets in bovine and healthy canine mitral valves showed a diffuse immunoreactivity for vW factor corresponding to the endocardial layer, and occasional cells in the spongiosa layer were positive to SMA (Figure 6A,B). Differently, the endocardial cells layer of the pathological valve presented a widespread positivity to vW factor (Figure 6C).

**Figure 7:**
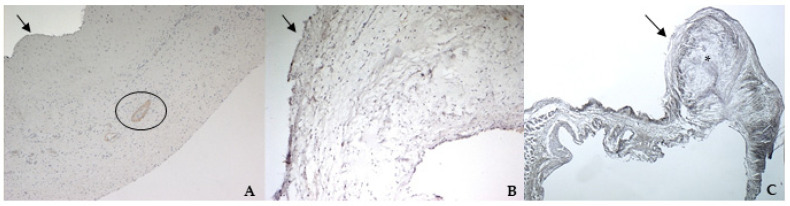
The picture shows positivity to Transforming Growth Factor (TGF) β1 in bovine (**A**) (magnification: 5×) and healthy (**B**) (magnification: 5×) and pathological (**C**) (magnification: 5×) canine mitral valves. Black arrows indicate the atrial aspect. In (A), the black circle delimitates a small artery in the spongiosa layer. *: area of myxomatous degeneration.

TGF β1 was identified in all specimens, with a stronger positivity in canine pathological valves compared to the bovine and the healthy canine valves (Figure 7A–C).

**Figure 8:**
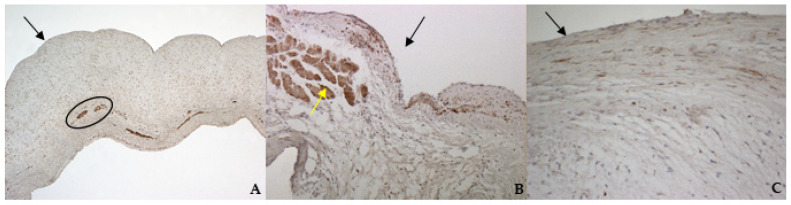
The picture shows positivity to Transient Receptor Potential Vanilloid 1 (TRPV1) in bovine (**A**) (magnification: 5×) and healthy (**B**) (magnification: 10×) and pathological (**C**) (magnification: 20×) canine mitral leaflets. Black arrows indicate the atrial aspect, while the yellow arrow indicates a strong positivity in myocardial fibers that normally penetrate into the proximal portion of the caudal leaflet. In (A), the black circle delimitates small arteries in the spongiosa layer.

TRPV1 positivity was considered ubiquitous in all samples: it was highlighted in endothelium, endocardium, and spongiosa and fibrosa layers in bovine and both types of canine valves (Figure 8A–C).

The negative controls did not show signs of immunoreactivity.

## 4. Discussion

The present study aimed to verify if an isolation method using a scraping technique could be efficient and reliable to obtain and cultivate VICs. To test the method, the authors decided to start with bovine hearts collected at a slaughterhouse, considering the easy availability and the size of the hearths. It was possible to appreciate a fibroblast-like cellular population few days after seeding, and ICC assays confirmed that only VICs were isolated and cultured. These results were considered encouraging; therefore, the same procedures were performed using normal and MMVD canine mitral valves. Viable cells were identified as a pure population of VICs and used for further characterization.

The novelty introduced in the described method consists in harvesting minced material obtained from scraping the atrial aspect of the mitral leaflet and seed it directly in uncoated Petri dishes, without prior enzymatic digestion using collagenase. Other studies were able to demonstrate the possibility to isolate VICs from a tissue section of 1–2 mm^2^ in culture plates, based on the migration of the cells [16,24]. Our method significantly reduces the difficulty of collecting material for VICs development in culture. Nevertheless, we used a high percentage of FBS (20%) to meet the cells’ metabolic requirements. Other authors [14] suggested that a high percentage of FBS could induce VICs activation from a fibroblast-like to a myofibroblast-like phenotype, with high expression of SMA and TGF β1; the same authors suggested that this is synonym of a pathological change of VICs. In that study, the cells were cultured in the presence of 2% FBS and of several kinase inhibitors, after enzymatic digestion. In our study, the experimental conditions were completely different, since we cultured the cells without any treatment, digestion, or inhibition and monitored at every passage the expression of VIM and SMA in order to verify that only VICs were cultured. We decreased up to 10% the concentration of FBS to perform proliferation assays and did not appreciate any variation in cell morphology even in the presence of VIM, SMA, vW factor, or TGF β1 (data not shown). It should be underlined that a sparse positivity to SMA is not necessarily correlated with a pathological process but is mostly related to the proliferation of cells. In in vitro conditions, cells normally proliferate when viable [8,25], and the laboratory conditions should be as close as possible to the in vivo ones [16].

In the present experimental design, we planned to perform both immunocytochemistry and immunofluorescence assays. According to the studies by Meier Bürgisser and colleagues [26,27], it is not possible to define a priori the best technique. These two papers compared different fixation (formalin and paraffin or cryopreservation) and staining (enzymatic or fluorescence immunohistochemistry) techniques. The results of their investigations are not unique and carefully describe the variations that can occur for certain markers and in some specific situations: the enzymatic technique could work better in identifying some structures or cells, but sometimes the outcome could be better by using immunofluorescence. According to the present literature, no technical notes or papers compared these two techniques in VICs before. We can conclude that in our specific context, the immunofluorescence technique could better identify the markers present in VICs.

According to the results obtained by SMA staining, in the bovine leaflet, a dense positivity was evident in arterioles wall and was sparsely present in the spongiosa and fibrosa layers. In the canine healthy leaflet, the positivity was evident in arterioles wall and in the fibrous and ventricular layers. In the canine pathological leaflet, SMA was more evident in the spongiosa layer of the proximal portion, whose normal structure was maintained. The positivity was also evident in the coaptation margin of the atrialis aspect, in the apical portion, closed to the degenerated area. This is compatible with the data presented in the literature, because SMA levels can increase in response to mechanical stimuli, that for the mitral valve are mainly due to a chronic coaptation of the leaflets [8,28].

The immunohistochemistry assays permitted to demonstrate some specific features of bovine and canine mitral valves and to study them in their leaflet structural context. All vimentin-stained sections showed a diffuse positivity, due to the large presence of mesenchymal cells. The bovine leaflet displayed a strong positivity in the spongiosa layer, which is the layer with the highest cell density, whereas in the fibrous layer, collagen is the major component. In the canine healthy leaflet, vimentin staining was more evident on the atrial and ventricular aspects, with a sparsely positivity in the fibrous and spongiosa layers. The canine pathological leaflet demonstrated a dense positivity in the spongiosa layer in the medial-apical portion, maintaining, however, a diffuse positivity.

The identification of TRPV1 was possible in both bovine and canine VICs using ICC and immunofluorescence. To the best of the authors’ knowledge, this is the first time that this identification is described in valvular interstitial cells. It must be noted that this identification was performed in cells derived from the last third of the leaflet. This specification is necessary since a wide heterogenicity of VICs was described according to the portion of the leaflet from which cells are collected (proximal or other parts) [28,29]. The expression of TRPV1 seemed to be stronger in canine pathological VICs compared to the physiological ones. These data open the possibility to investigate the role of this receptor in different conditions, such as in functional studies in the presence of specific ligands. We also performed IHC to evaluate the entire leaflet; TRPV1 appeared ubiquitous in both healthy and pathological valves. The presence of TRPV1 positivity in myocardial fibers, that normally penetrate into the first proximal portion of the caudal leaflet, and in the wall of small arterioles in the spongiosa layer is in accordance with Guinamard and Hof [21], who described the presence of TRPV1 in specimens of rat and mice myocardium and blood vessel wall. The presence of arterioles in the bovine mitral leaflets is in accordance with previous observations in physiological conditions [30]. Nevertheless, this is the first time that TRPV1 is identified using immunohistochemistry in the arterioles wall of bovine mitral leaflets.

The interest in canine MMVD is important not only for veterinary medicine but also for comparative pathology, because of some similarities shared by the human and the canine species [31]. VICs play an important role in the pathogenesis of the disease in human subjects, and their involvement in the same valvular disorders in canine species has been demonstrated [32].

A novelty of the present study is that the two canine mitral valves belonged to two dogs that underwent a complete echocardiographic exam prior to death. Measures and evaluations aimed to rank the hearts according to the ACVIM classification [6]. This is an innovative approach considering that none of the previous studies investigating VICs in canine species performed a similar enrollment but were limited only to the classification of morphological features according to the evaluation by Whitney [22]. This simple but fundamental step is necessary to move the research about VICs from an in vitro to an ex vivo approach and bring the bench closer to the clinical practice. Currently, the pharmacological treatment of MMVD in veterinary medicine involves the administration of drugs chosen depending on the severity of the disease according to the ACVIM guidelines [6], in order to enhance the quality of life and survival of the patients. These therapies are focused on limiting the symptoms and slowing down the progressive valve degeneration [3]. The need to find new therapeutic strategies must be oriented to a complete identification of the involved receptors. TRPV receptors are involved in a plethora of physiological and pathological conditions, which makes them promising pharmacological targets [33]. Some doubts remain about the feasibility of administering specific TRPV1 agonists and antagonists in vivo due to the high probability to induce side effects as a consequence of their wide distribution in different cells and tissues [19]. Some research groups are successfully evaluating a dietary integration of capsaicin, a partial TRPV1 agonist, in mice in order to reduce cardiac hypertrophy and fibrosis [11] and to ameliorate coronary function [34]. Therefore, it is crucial to understand the role of TRPV1 to treat pathological cardiac hypertrophy and other diseases using the current TRPV1 agonist or antagonist available in the pharmacopoeia [35]. Further studies are needed to confirm TRPV1 as a potential therapeutic target, since its presence in cardiac cells has been widely demonstrated, but nothing is known about its role and function in VICs. We are planning to investigate the relationship between TRPV1 and VICs in the development of MMVD in further studies.

## 5. Conclusions

In the present study, a reliable and easy-to-perform protocol to isolate and investigate bovine and canine VICs using an in vivo model is reported. Moreover, the identification through ICC, immunofluorescence, and IHC of TRPV1 receptor, both in bovine and canine species, is reported for the first time. Further experiments are necessary to understand the relationship between TRPV1 and VICs and their association with the development of MMVD and to hypothesize new therapeutic strategies.

## Figures and Tables

**Table 1:** Primary and the secondary antibodies and colorimetric detection kits used for immunocytochemistry (ICC), fluorescence, and immunohistochemistry (IHC).

Antibody	Reactive Species	Host Species and Clonality	Catalogue Number and Supplier	ICC Dilution	IHC Diluition	IHC Antigen Retrieval
**Primary Antibodies**						
anti-vimentin (VIM)	Pig	Mouse monoclonal	M0725 Dako	1:100	1:80	98 °C 30 min in Citrate Buffer, pH 6
anti-smooth muscle actin (SMA)	Human	Mouse monoclonal	M0851 Dako	1:200	1:300	none
anti-Von Willebrand Factor (vW Factor)	Human	Rabbit polyclonal	A0082 Dako	1:750	1:800	98 °C 30 min in Citrate Buffer, pH 6
anti-transforming growth factor β1 (TGF β1)	Human	Mouse monoclonal	sc-130348 Santa Cruz Biotech	1:50	1:50	98 °C 30 min in Tris-EDTA, pH 9
anti-vanilloid receptor 1 (TRPV1)	Rat	Mouse monoclonal	sc-398417 Santa Cruz Biotech	1:50	1:50	98 °C 30 min in Citrate Buffer, pH 6
**Secondary Antibody and Kit**						
Goat anti-Mouse IgG (H + L), AlexaFluor Plus 488	Mouse	Goat	A32723 Invitrogen	1:500		
Goat anti-Mouse IgG (H + L), AlexaFluor Plus 594	Mouse	Goat	A Invitrogen	1:500		
Dako REAL EnVision Detection System	Rabbit/Mouse		K5007 Dako			

## Data Availability

All data are reported in the present paper.
